# Traumatic aorta injuries in a rural area: late manifestations and review of therapeutic aspects

**DOI:** 10.1590/1677-5449.202200142

**Published:** 2023-05-12

**Authors:** José Maciel Caldas dos Reis, Flávio Roberto Cavalleiro de Macêdo Ribeiro, Adib Koury, Glauco dos Santos Melo, Murilo Vasconcelos de Oliveira, Vitor Hugo Guerreiro Américo Gomes, José Victor Figueiredo dos Santos, Sotero Gonçalves Sarquis

**Affiliations:** 1 Centro Universitário Metropolitano da Amazônia - UNIFAMAZ, Belém, PA, Brasil.; 2 Hospital de Clínicas Gaspar Vianna - HCGV, Belém, PA, Brasil.

**Keywords:** aorta, endovascular, thoracic aortic pseudoaneurysm, traumatic aortic injury

## Abstract

Traumatic thoracic aortic injuries (TTAI) are associated with high rates of morbidity and mortality. They are classified according to the extent of damage and computed tomography angiography has the highest sensitivity and specificity for identifying the degree of injury and potential associated lesions. Treatment strategies for TTAI are based on the type and extent of injury and associated lesions. The patient’s degree of stability can also help to define the choice of treatment, which can be conventional or endovascular surgery (EVAR) or even conservative management in selected cases. Among patients with adequate vascular anatomy, endovascular surgery is associated with better survival and fewer risks. The objective of this article is to describe a series of four cases followed up at a tertiary service in a Brazilian state that has few centers that provide high complexity care. Endovascular therapy was employed as the preferred method. All four patients had favorable outcomes, with no complications up to discharge, and are currently in outpatient follow-up.

## INTRODUCTION

Traumatic aortic injuries (TAIs) constitute a significant threat to life, because they are associated with high rates of morbidity and mortality and, statistically, are the second-ranked cause of death in traumas, only surpassed by intracranial hemorrhages.^1-3^ The most common etiology is blunt trauma, which is responsible for almost 90% of cases.^1^ Mortality can be as high as 80 to 85% and it is estimated that the majority of patients (85%) die at the scene of trauma.^4,5^ Those who are admitted to a hospital constitute a major challenge for the care team, since TTAI mortality during the first 24 hours can be as high as 30%, demanding rapid, effective, and consensual treatment.^5-8^

The terminology used by radiologists to describe TAIs can be confusing and inconsistent. A useful approach to considering the spectrum of injuries is a classification proposed by Starnes et al.,^9^ which describes four categories according to the extent of damage to the layers of the aorta wall, classified as: grade I - intimal tear; grade II - intramural hematoma; grade III - pseudoaneurysm (PAN); and grade IV - rupture.^8,9^ In turn, TTAI patients can be divided into two initial clinical presentation groups based on their hemodynamic status: unstable, with mortality rates exceeding 90%, and stable, with mortality less than 30%.^7-9^

The approach to treatment is based on rapid diagnosis and appropriate intervention. Treatment strategies vary by TTAI classification, the patient’s hemodynamic status, and presence of factors of severity or other associated lesions. The treatment options for TTAI include non-operative management, open repair, and endovascular intervention.^9-16^

As a result of the vast size of the state of Pará, considered the most populous state in the Brazilian Amazon and the second largest state in Brazil, compounded by a lack of specialists and high complexity centers, many cases of major vessel injury are recognized and treated late. We report a series of cases of late posttraumatic injuries to the thoracic aorta, seen at a tertiary hospital in the state capital Belém, and review their therapeutic aspects. The project was approved by the institution’s Research Ethics Committee (Ethics Appraisal Submission Certificate: 48063921.6.0000.0016; consolidated opinion number: 4,836,153).

## PART I: CLINICAL SITUATION

### Case 1

Patient ACP was a 22-year-old, brown-skinned, male, admitted to the Fundação Hospital de Clínicas Gaspar Vianna (FHCGV) after transfer from Altamira, Pará, having suffered a gunshot wound (GW) 32 days previously, leading to development of a PAN of the thoracic descending aorta. He was referred to the institution with a history of right thoracic drainage and radiological findings compatible with aortic involvement (PAN). At admission he was hemodynamically stable.

**Figure 1:**
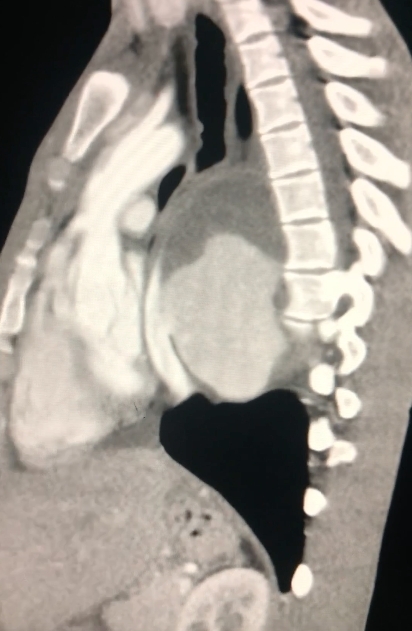
Diagnostic angiotomography showing grade III aortic injury III - case 1.

The echocardiogram report described normal baseline ejection fraction and vessel diameters. There was a retrocardiac formation compatible with hematoma/PAN of the descending thoracic aorta. Angiotomography showed a giant PAN of the thoracic descending aorta (Figure 1).

### Case 2

Patient ASC was a 74-year-old, brown-skinned male seen at the FHCGV after transfer from Anajás, Pará, having been the victim of a shotgun GW 37 days previously, presenting with multiple perforating injuries to the left hemithorax. He was referred to the institution with a history of left thoracic drainage and radiological findings compatible with cardiac and aortic involvement. The patient was in regular general health and hemodynamically stable.

**Figure 2:**
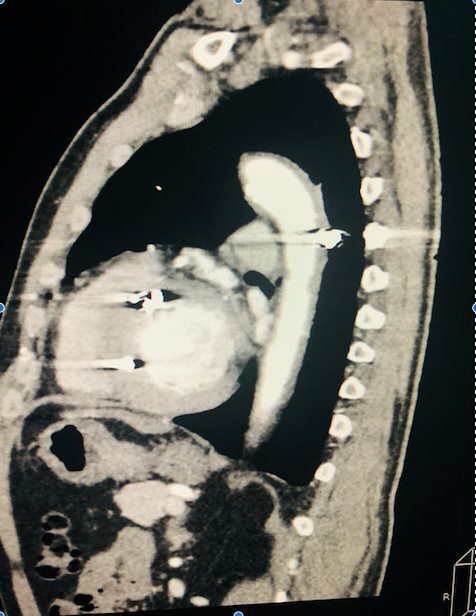
Diagnostic angiotomography showing grade III aortic injury - case 2.

An echocardiogram showed a retrocardiac formation compatible with hematoma of the thoracic descending aorta. Angiotomography showed a transmural lesion of the thoracic descending aorta with a contained hematoma (Figure 2).

### Case 3

Patient EMCF was a 19-year-old, brown-skinned male seen at the FHCGV after transfer from Paragominas, Pará, with a primary complaint of chest pain. He had been the victim of a stab wound (SW) 90 days previously and had a scar approximately 3 cm in length on the right dorsal paravertebral area. He was referred to the institution with radiological findings compatible with a widened mediastinum and suggestive of PAN of the thoracic descending aorta. On physical examination he was in regular general health and was hemodynamically stable.

Transthoracic echocardiogram showed evidence of a giant retrocardiac formation with mass effects (compression of the left atrium) and with intraluminal flow suggestive of a PAN of the thoracic descending aorta. Angiotomography showed a PAN of the thoracic descending aorta measuring 11.0 x 8.0 cm.

### Case 4

**Figure 3:**
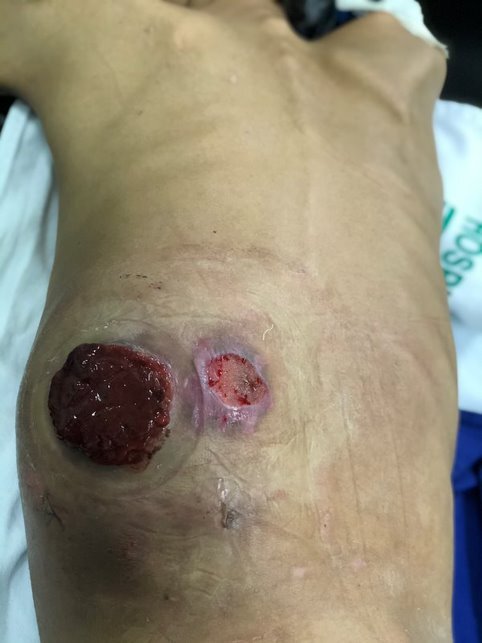
Mass effect of giant retroperitoneal pseudoaneurysm and hematoma at the thoracoabdominal transition wall - case 4.

Patient NRC was a 23-year-old, brown-skinned male seen at the FHCGV after transfer from Barcarena municipal district, Pará, after having suffered a fall from height (he was an açai palm worker) approximately 1 year previously, with progressive development of pain and swelling involving the left side of his back (Figure 3). He was referred to the institution after radiological findings compatible with involvement of the aorta at the thoracoabdominal transition, suggestive of PAN. On physical examination he was pale and emaciated, but hemodynamically stable.

**Figure 4:**
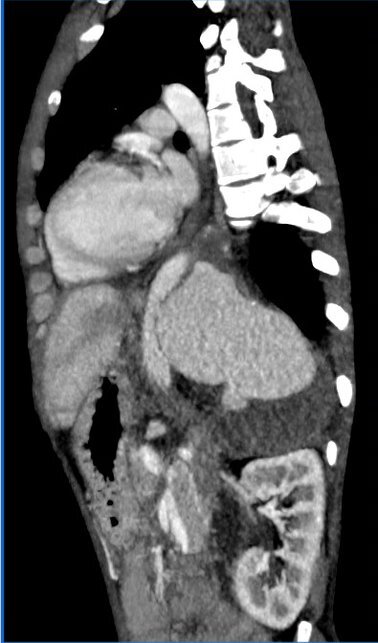
Diagnostic angiotomography with giant pseudoaneurysm (grade III injury) and large collection (hematoma) causing mass effects on the chest wall - case 4.

**Figure 5:**
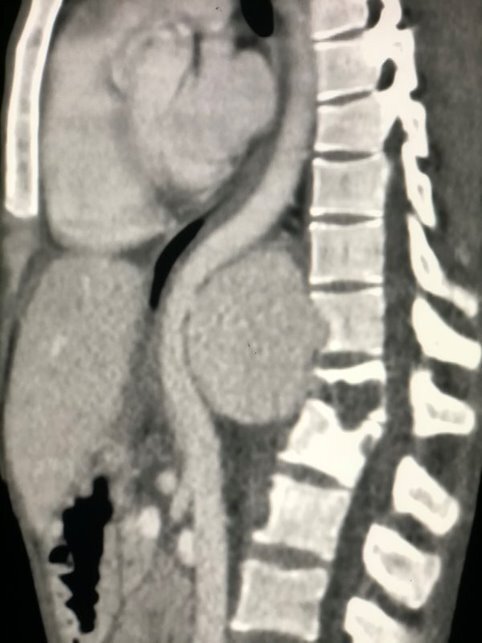
Diagnostic angiotomography showing the point of aortic injury at the thoracoabdominal transition - case 4.

Angiotomography of the thorax and abdomen showed a large-volume collection with vascular highlighting in the retroperitoneal space displacing the abdominal aorta anteriorly at the thoracoabdominal transition, with estimated constriction of 0.5 cm and estimated diameters of 18.7 x 9.7 cm along its longest transverse and anteroposterior axes, suggestive of a giant PAN of the thoracic descending aorta (Figures 4 and 5).

## PART II: WHAT WAS DONE

### Case 1

**Figure 6:**
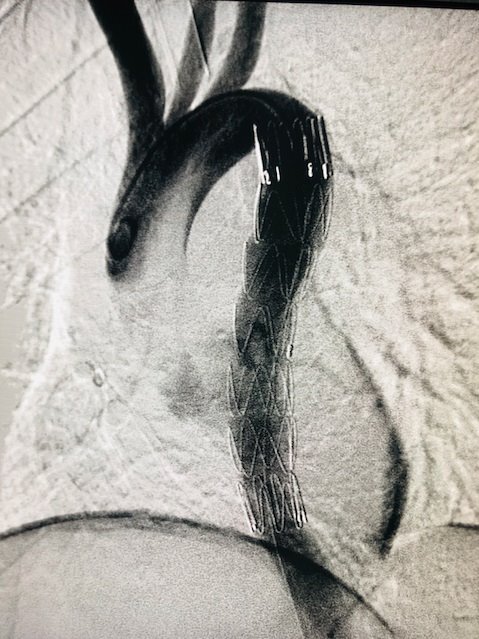
Endovascular repair of grade III aortic injury - case 1.

**Figure 7:**
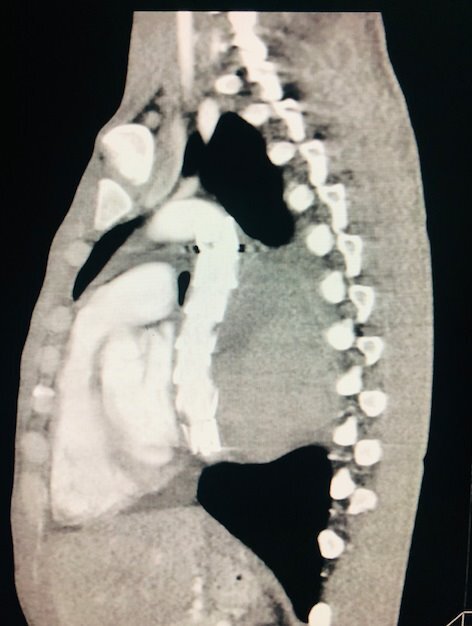
Control angiotomography 30 days after endovascular repair of grade III aortic injury - case 1.

On April 14, 2019, the patient underwent endovascular aneurysm repair with a 24/24/150 x 20 F Dominus® endoprosthesis (Braile, São Paulo, Brasil) via a right inguinal access (Figure 6). He spent 2 days in the intensive care unit (ICU) and was discharged from hospital five days after hospital admission, without complications. He remained in outpatient follow-up for 8 months. Control angiotomography at 30 days showed excellent sealing of the lesion (Figure 7). He is currently incarcerated.

### Case 2

**Figure 8:**
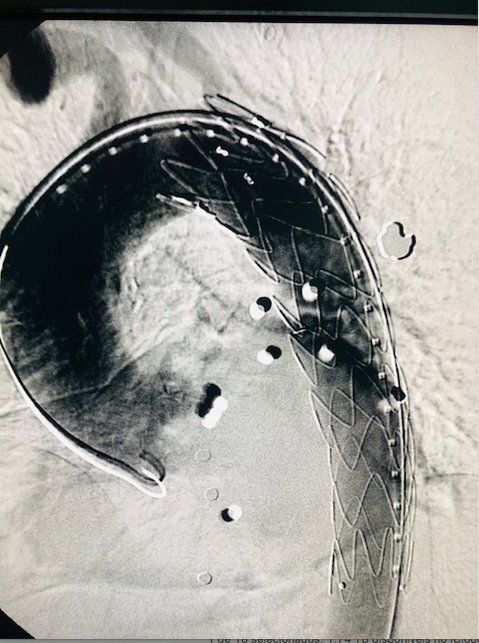
Endovascular repair of grade IV aortic injury - case 2.

On February 27, 2021, the patient underwent endovascular repair of the lesion with a 36/32/150 x 24 F Valiant Captivia® endoprosthesis (Medtronic, Minneapolis, EUA) via a right inguinal access (Figure 8). He spent 3 days in the ICU and was discharged from hospital, free from complications, on the sixth day after hospital admission. He remained in follow-up for 2 months until he returned to his municipal district of residence. Attempts to contact the patient and his family thereafter were unsuccessful.

### Case 3

**Figure 9:**
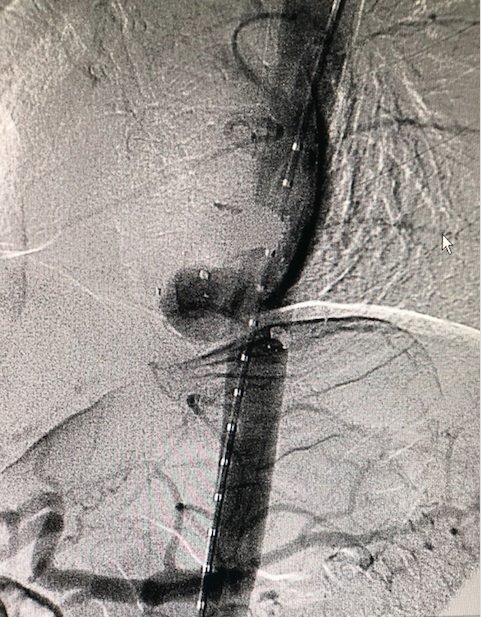
Control arteriography showing grade III aortic injury- case 3.

**Figure 10:**
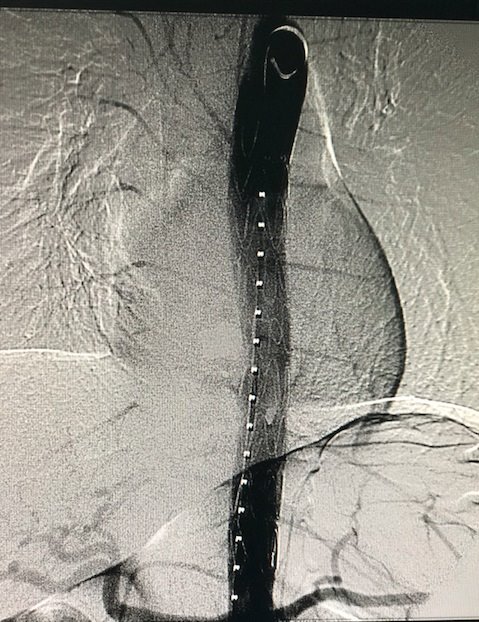
Endovascular repair of grade III aortic injury - case 3.

On February 21, 2021, the patient underwent endovascular repair of the PAN with a 22/22/150 x 20 F Valiant Captivia® endoprosthesis (Medtronic, Minneapolis, EUA) via a right inguinal access (Figures 9 and 10). He spent 24 hours in the ICU and was discharged from hospital after four days in hospital. He remains in outpatient follow-up.

### Case 4

**Figure 11:**
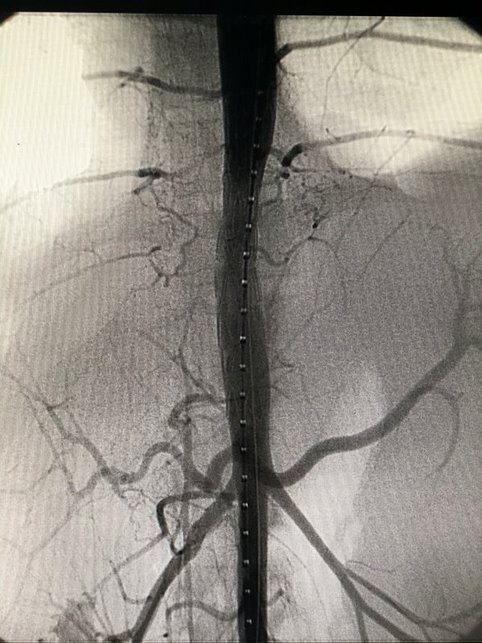
Endovascular repair of grade III aortic injury - case 4.

**Figure 12:**
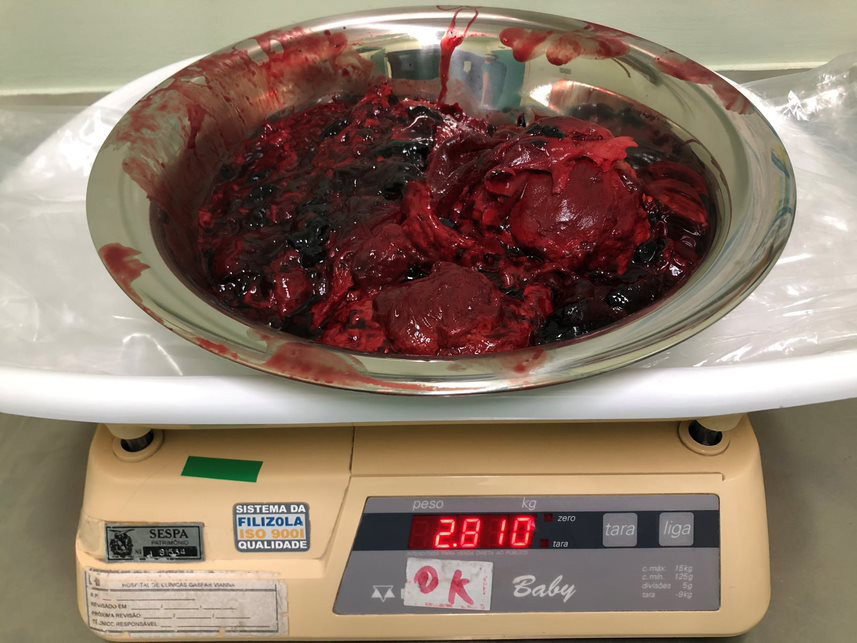
Content removed from the retroperitoneal space (giant hematoma) after endovascular repair of the injury- case 4.

On August 5, 2021, the patient underwent endovascular repair of the lesion with a 24/24/110 x 18 F Dominus® endoprosthesis (Braile, São Paulo, Brasil) via a right inguinal access (Figure 11). He spent 13 days in the ICU, undergoing drainage and emptying of the retroperitoneal hematoma, via a lumbar access, on August 13, 2021 (Figure 12). He was discharged from hospital 28 days after hospital admission and remains in outpatient follow-up.

Table 1 summarizes the cases by municipal district of origin, mechanism and grade of injury, the interval between trauma and treatment, the dimensions of the aorta, the endoprosthesis employed, and any postoperative complications.

**Table 1:** Descriptions of cases by municipal district of origin, mechanism of injury, interval from trauma to treatment, degree of injury, and dimensions of the aorta and the endoprostheses.

**Cases**	**Municipal district of origin (Pará)**	**Mechanism**	**Interval from trauma to treatment (days)**	**Aortic injury grade**	**Proximal diameter (mm)**	**Distal diameter (mm)**	**Device used**	**Postoperative complications**
**Case 1**	Altamira	GW	42	III	20	20	Dominus® (Braile, São Paulo, Brasil) 24/24/150 20 F	None
**Case 2**	Anajás	GW	47	IV	27	28	Valiant Captivia® (Medtronic, Minneapolis, EUA) 36/32/150 24 F	None
**Case 3**	Paragominas	SW	95	III	20	20	Valiant Captivia® (Medtronic, Minneapolis, EUA) 22/22/150 20 F	None
**Case 4**	Barcarena	FFH	369	III	22	20	Dominus® (Braile, São Paulo, Brasil) 24/24/110 18 F	None

GW: gunshot wound; SW: stab wound; FFH: fall from height.

## DISCUSSION

Traumatic thoracic aortic injuries occur in approximately 1 to 2% of trauma victims.^17^ However, few mechanisms of injury compare in lethality to acute aortic trauma, which translates to elevated mortality of up to 90% of patients, the great majority of whom do not even survive long enough to receive care in a hospital setting.^3,18,19^

The majority of TAIs occur distal of the left subclavian artery (in 80 to 95% of cases) and the traumatic mechanisms of high velocity or fall from height can be the results of a synchronic combination of both deceleration and the influence of shear and crushing forces.^17-20^

Therapeutic strategies for TTAI are conventional surgery (open repair), endovascular treatment (endovascular thoracic aorta repair, EVTAR) or conservative management (without surgery) in selected cases. Recently, combined (hybrid) interventions have also been adopted.^12,19,20^

Non-operative management of TTAI is currently recommended for patients with grade I injuries, because they are low risk and the majority of injuries heal spontaneously or remain stable.^10^ Only some patients with grade II TTAI will benefit from conservative management. There is uncertainty with regard to the likelihood of these injuries progressing to an intimal injury with rupture and of progression of chronic dissection, PAN, or even aortic rupture. As a result, defining the ideal time to treat is generally part of a complex and interdisciplinary decision-making process. If non-operative management is chosen, patients will require rigorous follow-up over the long term, until there is radiographic evidence that the injury is resolved.^4,10^

Current guidelines indicate that the preferred therapeutic management for grades II, III, and IV TTAI is endovascular repair, because EVTAR is associated with significantly lower rates of paraplegia, stroke, and death.^8,21,22^

Open TTAI repair is reserved for patients with anatomic conditions that rule out use of EVTAR, with ascending aorta involvement, or in certain situations in which open thoracic surgery is planned to treat other associated injuries.^3,8,21,22^

The literature is still contradictory with regard to the exact time to intervene. Society for Vascular Surgery guidelines recommend that intervention should be as soon as possible (preferably in < 24 hours).^4^ However, a study published in 2021 that analyzed data on 2,821 patients from an American trauma database and adjusted for degree of severity reported significantly better results in cases of EVTAR performed after 24 hours.^7^ Therefore, in patients who are hemodynamically stable, EVTAR should be performed electively after 24 hours.^4^ In contrast, unstable patients should be taken for immediate intervention.^3,4^

With current growth in use of EVTAR, it has been shown that intravascular ultrasound (IVUS) has an important role to play in assessment of selected patients with suspected TTAI.^23^ The main reason is the age of these patients, since the majority are young (three out of four patients in the cases in the present sample were under 25 years old). These patients have a healthy and elastic aorta wall, which can help with choosing the most appropriate device diameter, since it enables dynamic assessment of aortic diameters in real time.^8,15,23,24^

However, even though it is now considered a first-line treatment (evidence level IC)^8^ and is minimally invasive for patients with TTAI whose anatomy is appropriate, repair by EVTAR is still subject to potential risks related to the endoprosthesis.^8,21^

In 2008, Hoffer et al.^25^ conducted a systematic review comparing the conventional procedure to endoprosthesis in trauma cases involving the thoracic aorta, observing significantly lower mortality (9.7% vs. 27.7%; p < 0.001) and trends to fewer paralysis-related events (0.4% vs. 2.9%) and strokes (0.4% vs. 2.3%). Complications were similar in both groups. Patients who underwent endovascular interventions exhibited fewer systemic complications than conventional repair patients, but reported incidence of endoleak of up to 5.2%, predominantly type I.^25^ Other authors identified endoleaks as the principal complication of endovascular treatment, estimated at up to 15%.^3,4^

All four of the cases described in this article underwent late endovascular repair, despite having radiological signs of advanced and threatening injuries, because the patients had been traumatized in places that lack the infrastructure needed for rapid diagnosis and treatment of TTAI. After referral and transfer, they underwent endovascular repair and all outcomes were positive. There were no significant complications, they spent brief periods in hospital, and are in outpatient follow-up.

We conclude that TTAI is a condition that can cause high rates of lethality at the site of the traumatic event or even in hospital. In the majority of cases, rapid intervention is needed, but repair is possible even in cases that occur in rural areas and are treated late, preferably with endovascular techniques, enabling greater flexibility in terms of the time from admission to treatment and with high rates of clinical success, low morbidity, reduced surgical aggression, and shorter length of hospital stay.
