# Supplementary material for: Traumatic aorta injuries in a rural area: late manifestations and review of therapeutic aspects
Source: J Vasc Bras. 2023 May 12;22:e20220014. doi: 10.1590/1677-5449.202200142 (PMC10281037; doi:10.1590/1677-5449.202200142)
Supplement: Translation

# Translation: Lesões traumáticas de aorta em zona rural: série de casos com manifestações tardias e revisão dos aspectos terapêuticos

**DOI:** 10.1590/1677-5449.202200141

**Authors:** José Maciel Caldas dos Reis, Flávio Roberto Cavalleiro de Macêdo Ribeiro, Adib Koury, Glauco dos Santos Melo, Murilo Vasconcelos de Oliveira, Vitor Hugo Guerreiro Américo Gomes, José Victor Figueiredo dos Santos, Sotero Gonçalves Sarquis

**Affiliations:** 1 Centro Universitário Metropolitano da Amazônia - UNIFAMAZ, Belém, PA, Brasil.; 2 Hospital de Clínicas Gaspar Vianna - HCGV, Belém, PA, Brasil.

**Keywords:** aorta, endovascular, pseudoaneurisma da aorta torácica, lesão traumática da aorta

## Abstract

As lesões traumáticas da aorta (LTA) torácica estão associadas a altas taxas de morbimortalidade. São classificadas de acordo com a extensão do dano, e a angiotomografia computadorizada tem as maiores sensibilidade e especificidade para identificar o grau de lesão e potenciais lesões associadas. As estratégias terapêuticas para LTA são baseadas no tipo de lesão, na extensão e nas lesões associadas. Pode auxiliar na definição de conduta também o grau de estabilidade do paciente, podendo ser manejo cirúrgico convencional, endovascular (TEVAR) ou conservador em casos selecionados. Entre os pacientes com anatomia vascular adequada, a cirurgia endovascular está associada a melhor sobrevida e a menos riscos. O objetivo deste artigo foi descrever uma série de quatro casos acompanhados em serviço terciário, em um estado com poucos serviços de alta complexidade. A terapêutica endovascular foi empregada como método preferencial. Os pacientes apresentaram evolução favorável sem complicações até a alta e encontram-se em acompanhamento ambulatorial.

## INTRODUÇÃO

As lesões traumáticas da aorta (LTAs) representam uma importante ameaça à vida, pois estão associadas a altas taxas de morbimortalidade, sendo estatisticamente reconhecidas como a segunda causa de morte no trauma, atrás apenas das hemorragias intracranianas^1-3^ . A etiologia mais comum são os traumas fechados, sendo responsáveis por quase 90% dos casos^1^ . A taxa de mortalidade pode chegar a 80 a 85%, e estima-se que a maioria dos pacientes (85%) morre no local do atendimento pré-hospitalar^4,5^ . Os admitidos em hospital tornam-se um grande desafio para a assistência médica, pois a mortalidade da LTA nas primeiras 24 horas chega a 30%, o que exige, portanto, um tratamento rápido, eficaz e consensual^5-8^ .

A terminologia usada pelos radiologistas nas LTAs pode ser confusa e inconsistente. Uma abordagem útil, ao se considerar o espectro das lesões, é a classificação proposta por Starnes et al.^9^ , que descrevem quatro categorias de acordo com a extensão do dano sobre as camadas da parede da aorta, sendo classificadas em: grau I - laceração da íntima; grau II - hematoma intramural; grau III - pseudoaneurisma (PAN); e grau IV - ruptura^8,9^ . Quanto à apresentação clínica da LTA, pode-se dividir os pacientes em dois grupos iniciais conforme seu estado hemodinâmico: instáveis, que apresentam taxa de mortalidade superior a 90%, e estáveis, com mortalidade menor que 30%^7-9^ .

A abordagem terapêutica baseia-se no diagnóstico rápido e na intervenção adequada. As estratégias de tratamento variam conforme a classificação da LTA, do estado hemodinâmico do paciente e da existência de fatores de gravidade ou outras lesões associadas. Assim, as opções terapêuticas para LTA incluem manejo não operatório, reparo aberto e intervenção endovascular^9-16^ .

Portanto, em virtude da vastidão geográfica do estado do Pará, considerado o estado mais populoso da Amazônia brasileira e o segundo maior do Brasil, somada à carência de especialistas e centros com alta complexidade, muitos casos de lesões de grandes vasos são reconhecidos e tratados em fases tardias. Relatamos uma série de casos de lesões tardias pós-traumáticas de aorta torácica acompanhados em hospital terciário em Belém e revisamos os aspectos terapêuticos. O trabalho foi aprovado pelo Comitê de Ética em Pesquisa da instituição (Certificado de Apresentação de Apreciação Ética: 48063921.6.0000.0016; parecer consubstanciado: 4.836.153).

## PARTE I: SITUAÇÃO CLINICA

### Caso 1

Paciente ACP do sexo masculino, 22 anos, pardo, admitido na Fundação Hospital de Clínicas Gaspar Vianna (FHCGV), proveniente de Altamira, no Pará, tendo sido vítima de ferimento por projétil de arma de fogo (FPAF) com 32 dias de evolução, apresentando PAN de aorta torácica descendente. Veio referenciado à instituição com histórico de drenagem de torácica à direita e achados radiológicos compatíveis com envolvimento aórtico (PAN). Na admissão, encontrava-se estável hemodinamicamente.

O laudo do ecocardiograma revelou fração de ejeção e diâmetros de vasos da base preservados. Havia presença de formação retrocardíaca compatível com hematoma/PAN de aorta torácica descendente. Na angiotomografia, foi visualizado PAN gigante em aorta torácica descendente (Figura 1).

**Figura 1:** Angiotomografia diagnóstica com lesão de aorta grau III - caso 1.

### Caso 2

Paciente ASC do sexo masculino, 74 anos, pardo, foi atendido na FHCGV, proveniente de Anajás, no Pará, tendo sido vítima de FPAF tipo espingarda com 37 dias de evolução, apresentando múltiplas lesões perfurantes em hemitórax à esquerda. Veio referenciado à instituição com histórico de drenagem torácica à esquerda e achados radiológicos compatíveis com envolvimento cardíaco e aórtico. O paciente apresentava-se em estado geral regular e estável hemodinamicamente.

O ecocardiograma evidenciou formação retrocardíaca compatível com hematoma de aorta torácica descendente. Na angiotomografia, foi visualizada lesão transmural de aorta torácica descendente com hematoma de contenção (Figura 2).

**Figura 2:** Angiotomografia diagnóstica com lesão cardíaca e de aorta grau III - caso 2.

### Caso 3

Paciente EMCF do sexo masculino, 19 anos, pardo, foi atendido na FHCGV, proveniente de Paragominas, no Pará, com queixa principal de dor torácica. Havia sido vítima de ferimento por arma branca (FAB) com 90 dias de evolução, apresentando cicatriz de aproximadamente 3 cm no dorso paravertebral à direita. Veio referenciado à instituição por achados radiológicos compatíveis com alargamento de mediastino e sugestivos de PAN de aorta torácica descendente. Ao exame físico, apresentava-se em estado geral regular e estável hemodinamicamente.

O ecocardiograma transtorácico evidenciou presença de gigante formação retrocardíaca com efeito de massa (compressão de átrio esquerdo) e com fluxo intraluminal sugestivo de PAN de aorta torácica descendente. Na angiotomografia, foi visualizado PAN de aorta torácica descendente, com 11,0 x 8,0 cm.

### Caso 4

Paciente NRC do sexo masculino, 23 anos, pardo, foi atendido na FHCGV, proveniente do município de Barcarena, no Pará, tendo sido vítima de queda de altura (açaizeiro) há aproximadamente 1 ano, evoluindo com dor e abaulamento crescente em região dorsal esquerda (Figura 3). Veio referenciado à instituição após exames radiológicos compatíveis com envolvimento aórtico de transição toracoabdominal, sugestivos de PAN. Ao exame físico, o paciente apresentava-se hipocorado e emagrecido, porém estável hemodinamicamente.

**Figura 3:** Efeito de massa do pseudoaneurisma gigante e hematoma retroperitonial na parede em transição toracoabdominal - caso 4.

A angiotomografia de tórax e abdome revelou volumosa coleção com realce vascular localizada em retroperitônio rechaçando anteriormente a aorta abdominal em transição toracoabdominal, com pertuito estimado de 0,5 cm; diâmetros estimados em 18,7 x 9,7 cm nos maiores eixos transverso e anteroposterior, sugestivos de PAN gigante de aorta torácica descendente (Figuras 4 e 5).

**Figura 4:** Angiotomografia diagnóstica com pseudoaneurisma gigante (lesão grau III) e volumosa coleção (hematoma) com efeito de massa na parede torácica - caso 4.

**Figura 5:** Angiotomografia diagnóstica evidenciando ponto da lesão aórtica na transição toracoabddominal - caso 4.

## PARTE II: O QUE FOI FEITO

### Caso 1

No dia 14/04/19, foi submetido a correção endovascular da lesão, com endoprótese Dominus® (Braile, São Paulo, Brasil) 24/24/150 x 20 F por acesso inguinal direito (Figura 6). Permaneceu 2 dias na unidade de terapia intensiva (UTI) e recebeu alta hospitalar no quinto dia de hospitalização, sem complicações. Permaneceu em seguimento ambulatorial por 8 meses. No controle angiotomográfico de 30 dias, apresentava ótimo selamento da lesão (Figura 7). Atualmente, encontra-se em regime carcerário.

**Figura 6:** Correção endovascular de lesão de aorta grau III - caso 1.

**Figura 7:** Angiotomografia de controle 30 dias pós-correção endovascular de lesão de aorta grau III - caso 1.

### Caso 2

No dia 27/02/21, foi submetido a correção endovascular da lesão com endoprótese Valiant Captivia® (Medtronic, Minneapolis, EUA) 36/32/150 x 24 F por acesso inguinal direito (Figura 8). Permaneceu 3 dias na UTI e recebeu alta hospitalar, sem complicações, no sexto dia de hospitalização. Permaneceu em acompanhamento por 2 meses até retorno para município de origem. Não houve sucesso no contato com o paciente e familiar.

**Figura 8:** Correção endovascular de lesão de aorta grau IV - caso 2.

### Caso 3

No dia 21/02/21, foi submetido a correção endovascular do PAN com endoprótese Valiant Captivia® (Medtronic, Minneapolis, EUA) 22/22/150 x 20 F por acesso inguinal direito (Figuras 9 e 10). Permaneceu 24 horas na UTI e recebeu alta hospitalar no quarto dia de internação. Segue em acompanhamento ambulatorial.

**Figura 9:** Arteriografia de controle com achado de lesão aórtica grau III - caso 3.

**Figura 10:** Correção endovascular de lesão de aorta grau III - caso 3.

### Caso 4

No dia 05/08/2021, foi submetido a correção endovascular da lesão com endoprótese Dominus® (Braile, São Paulo, Brasil) 24/24/110 x 18 F por acesso inguinal direito (Figura 11). Permaneceu 13 dias na UTI, sendo submetido a drenagem e esvaziamento de hematoma retroperitoneal, por acesso lombar, em 13/08/2021 (Figura 12). Recebeu alta hospitalar no 28º dia de hospitalização e permanece em seguimento ambulatorial.

**Figura 11:** Correção endovascular de lesão de aorta grau III - caso 4.

**Figura 12:** Conteúdo retirado no retroperitônio (hematoma gigante) após correção endovascular da lesão - caso 4.

A Tabela 1 resume os casos de acordo com o município de origem, o mecanismo e o grau da lesão, o intervalo entre trauma e tratamento, as dimensões da aorta, a endoprótese utilizada e as complicações pós-cirúrgicas.

**Tabela 1:** Descrição dos casos de acordo com o município de origem, o mecanismo e o intervalo entre trauma e tratamento, o grau de lesão, as dimensões da aorta e a endoprótese.

**Casos relatados**	**Município de origem (Pará)**	**Mecanismo**	**Intervalo entre trauma e tratamento (dias)**	**Grau da lesão aórtica**	**Diâmetro proximal (mm)**	**Diâmetro distal (mm)**	**Material utilizado**	**Complicações pós-cirúrgicas**
**Caso 1**	Altamira	FAF	42	III	20	20	Dominus® (Braile, São Paulo, Brasil) 24/24/150 20 F	Ausentes
**Caso 2**	Anajás	FAF	47	IV	27	28	Valiant Captivia® (Medtronic, Minneapolis, EUA) 36/32/150 24 F	Ausentes
**Caso 3**	Paragominas	FAB	95	III	20	20	Valiant Captivia® (Medtronic, Minneapolis, EUA) 22/22/150 20 F	Ausentes
**Caso 4**	Barcarena	QA	369	III	22	20	Dominus® (Braile, São Paulo, Brasil) 24/24/110 18 F	Ausentes

FAF: ferimento por arma de fogo; FAB: ferimento por arma branca; QA: queda de altura.

## DISCUSSÃO

A LTA ocorre em aproximadamente 1 a 2% dos pacientes vítima de trauma^17^ . No entanto, poucos mecanismos de lesão se comparam à letalidade do trauma aórtico agudo, que se traduz em alta mortalidade - até 90% das vítimas -, não permitindo sequer que a grande maioria das vítimas receba atendimento em ambiente intra-hospitalar^3,18,19^ .

A maioria das LTAs ocorre distalmente à artéria subclávia esquerda, em 80 a 95% dos casos, e os mecanismos traumáticos de alta velocidade ou queda de altura podem ser resultantes de uma combinação sincrônica tanto de desaceleração quanto de influência de forças de cisalhamento e esmagamento^17-20^ .

As estratégias terapêuticas para LTA são cirúrgica convencional (reparo aberto), endovascular (tratamento endovascular da aorta torácica, TEVAR) ou manejo conservador (não operatório) em casos selecionados. Na atualidade, a combinação de intervenções (híbrido) também tem sido adotada^12,19,20^ .

O manejo não operatório da LTA é recomendado, atualmente, em pacientes com lesões de grau I, pois são de baixo risco e a maioria dessas lesões cicatriza espontaneamente ou permanece estável^10^ . Apenas alguns pacientes com LTA grau II podem se beneficiar do manejo conservador. Há incertezas quanto à progressão dessa lesão para uma lesão intimal com ruptura e evolução para dissecção crônica, PAN ou mesmo ruptura da aorta. Assim, definir o melhor momento para a terapia geralmente faz parte de um processo de tomada de decisão complexo e interdisciplinar. Na hipótese de manejo não operatório, os pacientes requerem acompanhamento rigoroso de longo prazo até que a evidência radiográfica da resolução da lesão seja obtida^4,10^ .

As diretrizes atuais apontam o manejo terapêutico preferencial da LTA graus II, III e IV em direção ao reparo endovascular, pois o TEVAR está associado a taxas significativamente mais baixas de paraplegia, acidente vascular cerebral e mortalidade^8,21,22^ .

O reparo aberto para LTA fica reservado para pacientes com condições anatômicas que excluem TEVAR, com envolvimento da aorta ascendente ou em algumas situações de cirurgia torácica aberta planejada para tratar outras lesões associadas^3,8,21,22^ .

A literatura ainda diverge sobre o momento exato para intervenção. A *Society for Vascular Surgery* recomenda, em suas diretrizes, que isso deve ser feito o mais breve possível (preferencialmente < 24 horas)^4^ . No entanto, um estudo publicado em 2021 que analisou os dados de 2.821 pacientes do banco de dados de trauma americano relatou um resultado significativamente melhor com um grau de gravidade ajustado no caso de TEVAR após 24 horas^7^ . Assim, no caso de pacientes hemodinamicamente estáveis, o TEVAR deve ser realizado eletivamente após 24 horas^4^ . Por outro lado, pacientes instáveis devem ser levados para pronta intervenção^3,4^ .

Com o uso atual e crescente do TEVAR, o ultrassom intravascular (IVUS) tem sido defendido como tendo um papel adjuvante importante na avaliação de pacientes selecionados com suspeita de LTA^23^ . A razão principal recai sobre a idade dos pacientes, pois a maioria é jovem (três dos quatro na presente amostra tinham idade inferior a 25 anos). Esses pacientes apresentam parede aórtica saudável e elástica, o que pode auxiliar na escolha mais adequada do diâmetro de dispositivo, pois possibilita uma avaliação dinâmica e em tempo real dos diâmetros aórticos^8,15,23,24^ .

No entanto, mesmo sendo atualmente considerada um tratamento de primeira linha (nível de evidência IC)^8^ e pouco invasivo nos pacientes com LTA com anatomia adequada, a reparação por TEVAR apresenta potenciais riscos relacionados à endoprótese^8,21^ .

Hoffer et al.^25^ conduziram uma revisão sistemática em 2008, comparando o procedimento convencional ou a endoprótese nos casos de traumatismo envolvendo a aorta torácica, e observaram uma mortalidade significativamente reduzida (9,7% vs. 27,7%; p < 0,001) associada a uma tendência de diminuição de eventos relacionados a paralisia (0,4% vs. 2,9%) e taxa de acidente vascular cerebral (0,4% vs. 2,3%). As complicações foram comparáveis entre os dois grupos. Os pacientes que passaram por intervenção endovascular apresentaram menos complicações sistêmicas em relação ao reparo convencional, mas referiram incidência de *endoleak* de até 5,2%, sendo o tipo I o predominante^25^ . Outros autores apontam o *endoleak* como a principal complicação na abordagem endovascular, com estimativas de até 15%^3,4^ .

No presente artigo, os quatro casos relatados foram submetidos à correção endovascular em fases tardias, mesmo apresentando aspecto radiológico preponderante de lesões de graus avançados ameaçadores, pois os pacientes sofreram trauma em localidade sem infraestrutura para rápido diagnóstico e tratamento de LTA. Após referenciados, passaram por correção pela técnica endovascular, e todos obtiveram desfechos positivos. Não houve complicações significativas, passaram por curto período de internação e permanecem em acompanhamento ambulatorial.

Concluímos que a LTA é uma condição que pode cursar com alta letalidade na localidade do evento traumático ou mesmo em ambiente intra-hospitalar. Requer, na maioria das vezes, rápida intervenção, mas com possibilidade de reparo mesmo em casos ocorridos em zona rural e tratados tardiamente, preferencialmente com a técnica endovascular, possibilitando uma maior flexibilidade no tempo de tratamento após a admissão e com alto sucesso clínico, baixa morbidade, menor agressão cirúrgica e menor tempo de internação hospitalar.
